# User Needs and Preferences for Multimodal Interaction in Social Robots for Later-Life Support: An Exploratory Survey and Conceptual Five-Layer Architecture

**DOI:** 10.3390/jintelligence14050085

**Published:** 2026-05-18

**Authors:** Ye Zhang, Yuqi Liu

**Affiliations:** 1 Academy of Interdisciplinary Studies, The Hong Kong University of Science and Technology, Hong Kong 999077, China; 2 School of Design, South China University of Technology, Guangzhou 510006, China; yuqiliu@scut.edu.cn

**Keywords:** user needs, multimodal interaction, social robots, later-life support, conceptual architecture, exploratory survey

## Abstract

Social robots hold promise for enhancing later-life support, but user needs and preferences for multimodal interaction modalities remain underexplored. This study explores awareness, willingness, perceived barriers, and modality–function associations across multiple interaction modalities among middle-aged and older adults, and proposes a conceptual five-layer architecture for design guidance. A questionnaire survey with 199 Chinese respondents (aged 45–64: 89.4%, 65+: 10.6%) examined perceptions of voice, visual, gestural, affective, sEMG, and brain–computer interface interactions. Voice and visual modalities were the most preferred; gesture and affective interactions were moderately accepted; awareness of sEMG was high but may reflect confusion with other sensor technologies; and BCI awareness and willingness were low. Based on survey findings and the literature, a conceptual five-layer architecture is presented to inform future social-robot design. The sample predominantly comprised middle-aged participants, so findings reflect prospective later-life users rather than the broader older-adult population. This study offers user-centered insights into multimodal social-robot interaction and provides design implications for future development rather than evaluating emotional-health interventions.

## 1. Introduction

As the global trend of population aging intensifies, leveraging technological support to enhance daily living, social engagement, and emotional well-being in later life has become a core societal concern (Zuo, 2025). Social robots, as intelligent assistive and companion tools, demonstrate potential in emotional companionship, health management, and social participation (Villafranca, 2022). However, traditional interaction methods, dominated by a single modality, struggle to address the challenges faced by the middle-aged and older users, including sensory decline, the digital divide, cognitive load, and contextual diversity. This often results in high barriers to robot usage and weak perceived emotional responsiveness (Cheung, 2021). Multimodal human–computer interaction integrates multiple perception and expression channels—including visual, voice, tactile, and facial expression recognition—to achieve redundancy, complementarity, and semantic coordination. This approach holds promise for enhancing usability and acceptability in complex home and care environments. Emotional intelligence (EI) offers a theory-grounded lens to explain why and how such multimodal social-robot interactions may support older adults’ socio-emotional functioning and emotion-related communication (Salovey & Mayer, 1989). In this paper, EI is used as a conceptual lens, referring to emotion perception, emotion understanding, and emotion regulation processes that are relevant to well-being in mid-to-later life (Gross, 2002; Gross et al., 1998). From a design perspective, social robots can be viewed not only as assistive tools but also as potential interaction systems that scaffold empathic communication and low-risk regulation-oriented support in everyday contexts.

Existing research has largely focused on technical feasibility or localized scenarios, with insufficient user-side evidence regarding Chinese middle-aged and older users’ perceptions and expectations of different interaction modalities and their implications for design related to psychological and social well-being. According to recent demographic projections by the World Health Organization (WHO) and the United Nations (UN), the global population aged 60 years and older is expected to double from 1.1 billion in 2020 to over 2.1 billion by 2050. Within this trajectory, adults currently in midlife (approximately 40–55 years) are expected to become a substantial segment of the future older population (*Ageing and health*, n.d.). As this cohort approaches later life, their current perspectives on technology, autonomy, and care preferences are anticipated to shape the design and adoption of aging-in-place systems and assistive technologies. This midlife stage is also relevant for understanding future care expectations, because emotion regulation strategies, technology familiarity, and attitudes toward care formed in midlife may influence later-life technology adoption and perceived support. Moreover, policy reports, such as the OECD’s “Preventing Aging Unequally,” emphasize the importance of addressing aging readiness not only among older adults but also among the middle-aged population (*Preventing ageing unequally*, 2017). Their behavioral patterns, digital literacy, and technological expectations serve as early indicators for future care preferences. In this study, we therefore regard middle-aged users as prospective later-life users—“proto-users”—whose current experiences and anticipations provide exploratory insight into the design of socially assistive robotics and multimodal interaction systems for future aging populations.

Older adults face psychosocial challenges such as loneliness, depression, and social isolation (Koh et al., 2021), and enhancing their mental health and social welfare is a societal consensus. Research indicates that social robots hold potential in companionship, cognitive stimulation, and care coordination. However, challenges persist in contextual adaptation, usability, and ethical management. Traditional later-life support systems, constrained by limited human resources and funding, struggle to effectively address the growing emotional and companionship needs of the aging population (Zhang, 2024). Meanwhile, the rapid advancement of artificial intelligence and robotics has opened new avenues for addressing the challenges of an aging population. Social robots, designed to interact with and communicate with humans, can assist with caregiving and companionship, thereby potentially improving the quality of life for older users (Søraa et al., 2023). Research has demonstrated that companion robots, pet-like robots, and humanoid robots can support mood, perceived companionship, and social engagement in some care and community settings. For instance, pet-like robots such as Paro have been associated with comfort, engagement, and stress-related outcomes in some older-adult care contexts through their zoomorphic form and tactile feedback (Bradwell et al., 2021). Human-like robots such as Pepper, equipped with voice and facial expression interaction capabilities, proactively engage with older users, remind them to take medication, and assist with simple daily activities (Liao et al., 2023). These systems are often designed to elicit positive affect and social engagement, suggesting potential pathways for supporting emotional well-being (e.g., companionship routines, empathic interaction, and reminders that reduce daily stress), although evidence across studies remains heterogeneous and context dependent. Moreover, companion robots like LOVOT have received positive user feedback in real-world applications. Taken together, these examples motivate an EI-informed interpretation of social robots as platforms that may support emotion-related processes (perception, empathy, and regulation scaffolding) through sustained multimodal interaction.

Research further indicates that intelligent technologies have significant potential to support older adults with cognitive impairments. For instance, Pollack et al. highlight how artificial intelligence can assist dementia patients in performing daily activities (Heerink et al., 2010). Social robots integrate artificial intelligence’s perception and decision-making capabilities, enabling them to communicate with users through multimodal channels such as voice, vision, and touch. They provide companionship, monitoring, and emotional support functions designed to address key needs among older adults and may help supplement overstretched caregiving resources, while also supporting mental health and social engagement through interactive companionship. Against this backdrop and in response to these challenges, this study proposes applying multimodal interaction to the field of social robots for later-life support, aiming to translate user needs, modality preferences, and perceived constraints into design-oriented interaction capabilities and system architecture considerations. Multimodal interaction refers to achieving natural human–machine communication through multiple communication channels such as voice, body movements, touch, and facial expressions (Oviatt, 1999). Oviatt’s seminal research demonstrates that carefully designed multimodal systems can leverage the strengths of each modality to create complementarity, enhancing the naturalness and robustness of interactions. For middle-aged and older users who may experience declining audiovisual abilities and limited experience with digital technology, multimodal interaction holds promise for delivering more user-friendly and accessible interfaces (Biswas & Samanta, 2008). For instance, combining voice commands with touchscreens can lower the operational threshold for older users interacting with social robots. The purpose of this study is to explore how multimodal interaction supports older adults’ socio-emotional functioning and perceived emotional support, with an EI-oriented focus on emotion perception, empathic responsiveness, and regulation-oriented assistance during social-robot interaction. At this stage, the study focuses on user demand, acceptability, and design implications rather than direct testing of emotional-health intervention outcomes.

This study aims to explore recognition, willingness to use, perceived barriers, and functional needs of middle-aged and older adults across six interaction modalities in social robots: visual interaction, voice interaction, somatosensory interaction, affective interaction, surface electromyography (sEMG), and brain–computer interface (BCI). Based on an exploratory questionnaire survey and the related literature, this study proposes a conceptual five-layer architecture for multimodal social robots in later-life support contexts. Unlike intervention studies, this research does not evaluate emotional-health outcomes or test the effectiveness of robot-based interventions. Instead, it focuses on user needs, modality preferences, perceived constraints, and design implications for future prototype development and empirical evaluation.

## 2. Literature Review

### 2.1. An Overview of Multimodal Interaction Research

Multimodal human–computer interaction represents a cutting-edge direction in current HCI research. Its core concept involves introducing multiple perception and expression channels—such as voice, touch, gestures, and vision—beyond single-modality interaction, thereby simulating the richness of natural human communication (Oviatt, 1999). Oviatt noted in her work that compared to traditional keyboard–mouse interfaces, multimodal interfaces offer users greater expressive power and naturalness (Oviatt, 1999). Her research demonstrates that well-designed multimodal systems can complement and integrate the strengths of different modalities, thereby enhancing interaction robustness. When one input mode falls short, another can compensate. For instance, while speech recognition may be affected by noise, integrating gestures or touch commands can improve the system’s accuracy in understanding user intent. With the maturation of AI technologies like speech recognition and computer vision, multimodal interaction systems have entered a practical phase. New-generation systems no longer rely solely on simple speech-plus-click combinations but can simultaneously process more complex signal combinations.

Multimodal interaction is particularly relevant for older users and later-life support contexts. On one hand, older adults often experience varying degrees of visual, auditory, or physical functional decline (Dias et al., 2012). A single interaction modality may not suit all elderly users—for instance, text-only interfaces are unfriendly to those with poor vision, while voice-only interfaces pose challenges for those with hearing loss. Multimodal interfaces, however, allow users to select the most comfortable interaction method or combine multiple methods based on their preferences and capabilities (El Kamali et al., 2023). In a study, Dias et al. developed a home multimedia system that supports multiple inputs, such as voice, touch, and gestures. Results showed that natural interactions combining voice and touch significantly improved elderly users’ success rates and satisfaction with services like email and chat, helping reduce feelings of digital isolation (Dias et al., 2012). On the other hand, multimodal interaction also provides richer channels for emotional communication. For elderly users seeking social companionship, combining a robot’s verbal content with nonverbal cues such as facial expressions and body movements creates a more vivid and intimate communication experience, thereby enhancing the robot’s “sociability” and “user affinity.” For instance, when a social robot offers comfort through voice chat while gently holding the user’s hand or nodding, it conveys care and understanding more effectively than words alone. This multimodal emotional expression partially mimics human interaction, potentially supporting perceived companionship among older users living alone (Wada et al., 2004).

However, despite its clear advantages, multimodal interaction still faces numerous design challenges. The first challenge lies in integrating and synchronizing information across modalities—specifically, enabling robots to understand and correlate inputs such as speech and vision, preventing “talking at cross-purposes.” Currently, researchers are focused on establishing unified multimodal semantic fusion models to analyze the consistency and complementarity of user commands across different modalities (Oviatt, 1999). Second, user preferences and habits vary across individuals, with significant differences observed among elderly populations (El Kamali et al., 2023). Consequently, multimodal systems require adaptive capabilities to learn user habits and adjust interaction strategies. For instance, some older users prefer voice communication, prompting systems to prioritize voice dialogue, while others may favor button-based touch interfaces. In such cases, systems should simplify voice input to prevent user burden. Finally, interface design must prioritize seamless modal transitions and fault-tolerance mechanisms. This ensures that when one method fails, users understand they can retry via another approach without abandoning the interaction (Oviatt, 1999). Overall, multimodal interaction offers novel approaches to enhancing elderly–computer interaction experiences. However, its effective application relies on in-depth user research and comprehensive system integration to fully realize its “user-centered” potential.

### 2.2. Emotional Intelligence as a Conceptual Lens for Social-Robot Emotional Support Design

Emotional intelligence (EI) provides a theoretically grounded lens for interpreting how social-robot interaction may support emotional health and socio-emotional functioning in later life. Classic EI scholarship distinguishes between ability EI and trait EI (also known as emotional self-efficacy). Ability EI conceptualizes EI as a set of cognitive-emotional abilities—typically including the capacity to perceive, use, understand, and manage emotions—and is commonly assessed using performance-based measures (e.g., MSCEIT). In contrast, trait EI emphasizes relatively stable self-perceptions and dispositions related to emotional functioning and is more often measured using self-report instruments (e.g., TEIQue, WLEIS) (Joseph & Newman, 2010; Mayer et al., 2003, 2008; Salovey & Mayer, 1989).

In the present study, EI is not treated as a single score to be directly measured in the current dataset; rather, it is used as a conceptual lens for understanding emotion-related interaction processes in social-robot design. Specifically, we focus on three EI-related processes that are highly relevant to social-robot design for older adults: emotion perception (e.g., detecting affective cues from voice, facial expressions, or interaction behavior), emotion understanding (e.g., interpreting emotional meaning in context through dialogue and interaction history), and emotion regulation support (e.g., prompting reappraisal, relaxation, or supportive routines). This framing is also consistent with process-oriented emotion regulation theory, which emphasizes how regulation strategies shape well-being and social functioning(Gross et al., 1998; Gross & John, 2003; Salovey & Mayer, 1989).

The relevance of EI to individual development is particularly salient in mid-to-later life. Research has linked EI-related capacities to subjective well-being, life satisfaction, and psychological adjustment (Y. Chen et al., 2016; Delhom et al., 2017; Sánchez-Álvarez et al., 2016), and some studies suggest that EI may partially mediate associations between age and well-being outcomes (Mattingly & Kraiger, 2019). Although the magnitude and direction of these relationships vary across measures and populations, this literature supports the use of EI as a theoretically meaningful lens for examining how emotional-support technologies may influence later-life adaptation and socio-emotional functioning (Abdi et al., 2018; Kachouie et al., 2014; Pu et al., 2019).

From a social-robot design perspective, EI helps organize the conceptual relationships among emotion-related processes, interaction components, and potential future evaluation outcomes. Multimodal channels (e.g., voice, visual cues, touch/gesture, and affective interaction signals) may support EI-relevant processes (perception, understanding, and regulation scaffolding), which in turn can be operationalized as intervention components such as empathic conversational responses, emotional check-ins, reflective prompts, social-connection nudges, and calming routines (Pu et al., 2019). Future prototype-based studies may examine whether such components are associated with perceived social support, reduced loneliness, or improved emotional well-being. Importantly, in the current study, this chain is used as a design and evaluation scaffold, not as evidence of the efficacy of causal intervention (Mehrabi & Ghezelbash, 2025; Yen et al., 2024).

This cautious positioning is warranted because evidence on social robots and emotional outcomes in older adults, while promising, remains heterogeneous. Systematic reviews and meta-analyses have reported potential benefits for outcomes such as loneliness and depression, particularly in certain care contexts (e.g., long-term care, structured interventions), but findings vary by robot type, intervention duration, comparator conditions, sample size, and outcome measurement choices (Abdi et al., 2018; Yen et al., 2024). Therefore, we adopt EI as a theory-informed lens to guide the conceptualization, mapping, and prioritization of multimodal social-robot capabilities, while reserving direct tests of the effectiveness of emotional-health interventions for future longitudinal or controlled studies.

For the purposes of this paper, this EI-informed lens serves three functions: (1) it clarifies why affective and multimodal interaction features are central to elderly-oriented social robots beyond usability alone; (2) it supports the interpretation of questionnaire findings as demand- and constraint-side evidence for EI-relevant interaction capabilities (rather than treatment effects); and (3) it provides a conceptual bridge from user needs to the proposed five-layer multimodal interaction framework, which can support future measurement development and intervention evaluation planning.

### 2.3. Social Robot Concept and Role Positioning

Social robots are generally defined as autonomous or semi-autonomous robotic systems capable of engaging in socially meaningful behaviors and affective interactions with humans (Fong et al., 2003). Unlike traditional robots that primarily emphasize industrial productivity or precision task execution, social robots place greater emphasis on communication, collaboration, and relationship-building in human-centered contexts (Dautenhahn, 2007). Existing definitions vary in emphasis across research communities. For example, Fong et al. describe social robots as embodied agents embedded in heterogeneous social groups composed of humans and robots, with capabilities for mutual recognition, social interaction, communication, and learning based on prior experience (Fong et al., 2003). This perspective highlights several core attributes relevant to social robots: embodiment, identity recognition, social interaction competence, memory, and adaptive learning. Accordingly, social robots are not only command-execution tools, but also social actors designed to participate in ongoing human-centered interactions.

Depending on their functions and application scenarios, social robots can fulfill a diverse range of roles in society. Dautenhahn identified three robot roles in therapeutic projects for children with autism: therapeutic playmates (play partners who engage children to stimulate social behaviors), social mediators (facilitators of human-child interactions, such as guiding children to interact with therapists), and social modeling agents (demonstration models guiding children to imitate social behaviors) (Dautenhahn, 2003). These roles extend beyond special education, offering insights for general human–robot interaction. In later-life support or care contexts, for instance, social robots can serve as companions—alleviating loneliness through conversation or singing—assistants—reminding older users to take medication or providing emergency alerts—or social facilitators—helping older users engage in social activities or connect with family (Sparrow & Sparrow, 2006). Fong et al.’s review of social robots also categorizes them into companionship robots (providing emotional support), service robots (assisting daily tasks), and educational/rehabilitation robots (interacting with users in cognitive training and physical rehabilitation) (Cabibihan et al., 2013; Fong et al., 2003). In elderly care settings, companion robots (e.g., bionic pets, conversational companions) and service robots (e.g., delivery robots, telepresence robots) are the two most prevalent types (Cabibihan et al., 2013; Casiddu et al., 2015).

In addition to functional classification, the social acceptability of robots depends on whether their behaviors align with human social expectations. Dautenhahn emphasized that social robots should follow social etiquette (sometimes described as “robotiquette”) to facilitate smooth integration into human environments. Examples include maintaining appropriate interpersonal distance, respecting personal space, and producing movements and vocalizations that are comfortable and credible to users (Dautenhahn, 2007). Related work has therefore focused on equipping robots with socially intelligible behaviors, including emotion expression, adaptive communication styles, and context-sensitive interaction strategies. Breazeal’s notion of “approachable robots,” for instance, underscores the importance of expressive behaviors (e.g., facial expression, prosody, and gesture) in shaping users’ perceptions of robots as social rather than purely mechanical agents (Heerink et al., 2010). Prior research also suggests that adherence to familiar social norms (e.g., greeting, thanking, and turn-taking) can facilitate acceptance, whereas norm violations or unnatural behavior may reduce trust and, in some cases, evoke discomfort associated with the uncanny valley (Dautenhahn, 2007). These considerations are especially important in the design of social robots for older adults, whose communication preferences and social norms may differ from those of younger users. Design choices such as speech rate, tone, politeness conventions, and interaction pacing may substantially influence perceived respect, comfort, and willingness to engage. Therefore, role positioning for older-adult-oriented social robots should not be defined solely by technical capability, but also by how well robot behaviors align with users’ social expectations, dignity, and emotional needs.

Overall, social robots combine technical and social attributes. Technically, they depend on AI-enabled capabilities for perception, inference, and action; socially, they must remain compatible with human psychological expectations and interaction norms. In older adult care, examples such as Paro and Pepper illustrate different balances between emotional companionship and practical assistance (Wada et al., 2004). Paro, a pet-like robot, emphasizes tactile and affective interaction to support comfort and engagement, whereas Pepper is more commonly positioned as a service assistant and conversational partner, using speech, gesture, and expressive behavior to provide information and social interaction (Wada et al., 2004). Regardless of form factor or modality configuration, the role positioning of social robots in later-life support contexts should be framed as supportive rather than substitutive: they may augment care and companionship, but they should not be conceptualized as complete replacements for human caregivers. This positioning is particularly important for minimizing ethical risks and preserving human dignity in emotionally sensitive care contexts (Sparrow & Sparrow, 2006).

### 2.4. Older Adults’ Needs, Acceptance, and Interaction Preferences

As a key target user group for social robots, older adults warrant an in-depth investigation of their specific needs and patterns of technology acceptance. Prior studies suggest that older adults differ from younger adults in physiological functioning, cognitive abilities, and daily routines, resulting in more diverse and individualized requirements for human–robot interaction (El Kamali et al., 2023). First, older adults often place particular emphasis on safety and independent living. One study (2014) noted the value of technologies that support independent living among older adults with disabilities (Agree, 2014). Consequently, many older users seek assistance from social robots in daily routines, such as medication reminders and fall detection, to enhance their sense of security while living independently at home. Simultaneously, emotional companionship is important for older adults. As societal aging intensifies and the number of older adults living alone or with limited family contact increases, many face shrinking social circles and emotional isolation, and hope robots can provide companionship, conversation, and psychological comfort. For instance, Søraa’s research found that elderly respondents hoped robots could understand them, remember their preferences, and accompany them like friends; yet they also worried about robots exercising excessive control or invading their privacy (Søraa et al., 2023). These findings suggest that older adults seek technologies that are both useful and emotionally supportive, while still respecting privacy and dignity.

Multiple factors influence the acceptance of social robots among older adults. The Traditional Technology Acceptance Model (TAM) indicates that perceived usefulness and ease of use are key determinants of users’ willingness to adopt the technology. These principles remain applicable to older adults: robots must offer practical functionality and straightforward operation to motivate use among older users (Heerink et al., 2010). Experiments by Heerink et al. indicate that factors such as social presence and trust are particularly crucial. In other words, if older users perceive the robot as providing enjoyable, interactive experiences and believe it is reliable and safe, they are more likely to continue using it (Heerink et al., 2010). Additionally, Flandorfer’s research highlights that demographic factors like age, education level, and prior technological experience also influence acceptance. Technology adoption among older users is not uniform: older adults often prioritize ease of use more than younger users, and functional preferences differ between men and women. Developers must therefore consider the needs of distinct demographic segments (Flandorfer, 2012). Additional research has examined the NAO humanoid robot in the context of later-life support. For example, a pilot study showed that robot-mediated activity using the NAO robot could support health-promotion communication and engagement among older adults in geriatric facilities (Blavette et al., 2022).

The preferences of older users regarding specific functions and interaction methods of social robots also warrant attention. Research indicates that older adults generally prefer robots to focus on everyday topics rather than overly technical guidance. In terms of tone and intonation, robots should maintain politeness and patience while avoiding overly rapid or complex speech patterns (Søraa et al., 2023). Regarding interaction methods, some older users prefer voice-based conversations because they resemble human-to-human communication and require no learning of complex operations, while others prefer physical buttons or touchscreens, perceiving them as more intuitive and reliable (El Kamali et al., 2023). Nomura and Nakao’s study compared how older and younger adults interpret robots’ emotional gestures, revealing lower recognition rates among older users for certain emotional expressions. This suggests robots should avoid subtle or ambiguous body language when conveying emotions to older users, potentially combining verbal explanations to prevent misunderstandings (Nomura & Nakao, 2010). Frennert and Östlund surveyed 126 older adults in the UK and identified seven major concerns about social robots, including privacy, safety, social isolation, and dehumanization. For instance, some older users worry that robots recording their behavioral data could lead to privacy breaches, or that over-reliance on robotic companionship might reduce opportunities for real-person social interaction (Frennert & Östlund, 2014). These concerns remind designers to strike a balance between technological implementation and human-centered care. This includes implementing data protection measures and encouraging robots to guide users to interact with others rather than completely replace human socialization.

Additionally, older adults’ acceptance of social robots can evolve with actual usage experience. Upon initial exposure to robots, some older users may experience technological anxiety or resistance, believing they cannot learn to use them or do not need them (Søraa et al., 2023). However, research indicates that after a period of interactive engagement, older users’ attitudes often become more positive. Provided the robots are easy to operate and offer tangible benefits, they gradually build trust and integrate them into their daily lives (Wada et al., 2004). For instance, in a 2017 study by Abdollahi et al., elderly individuals with depressive tendencies participated in a companion robot trial. Results revealed that participants not only accepted the robot as a daily companion but also maintained a high interest in using it weeks later, without abandoning it after the novelty wore off (Abdollahi et al., 2017). This demonstrates that genuinely involving target users in technology trials and ensuring positive experiences are crucial for enhancing long-term acceptance. Some scholars advocate adopting co-creation or participatory design methodologies early in the development process, inviting elderly users and caregivers to jointly design robot functions and interfaces (Darriba Frederiks et al., 2019). This user involvement helps ensure product alignment with actual needs while emphasizing user identification with the product, thereby increasing the likelihood of future adoption.

### 2.5. Research Gaps and Positioning of the Present Study

Despite the promising potential of social robots and multimodal interaction in older-adult care, the existing literature still shows several important gaps that limit translation into robust, user-centered design frameworks. First, much prior work remains technology-driven, with relatively limited attention to user-side evidence on older adults’ lived experiences, emotional expectations, and interaction constraints (Heerink et al., 2010; Søraa et al., 2023). Many studies emphasize improvements in robot functionality or system performance (e.g., speech recognition accuracy or navigation optimization) while giving comparatively less attention to how older adults subjectively experience these systems during real use (Giuliani et al., n.d.). In addition, some human–robot interaction research in aging contexts still relies heavily on developer assumptions about user needs, with insufficient direct participation from older adults in early-stage requirement elicitation (Oudshoorn & Rommes, 2004). This gap can contribute to designs that are technically capable but insufficiently aligned with everyday routines, emotional needs, and adoption conditions.

**Figure 1:**
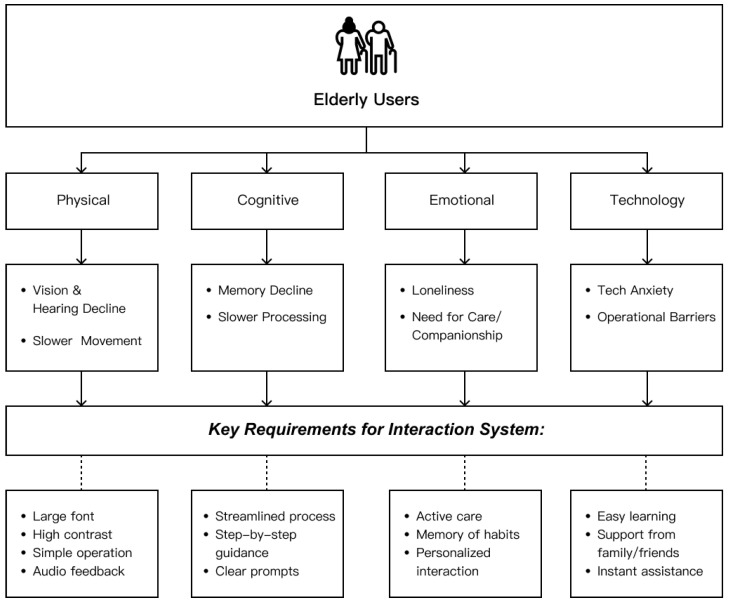
Characteristics of older users and key requirements for multimodal human–robot interaction. The schema maps age-related physical, cognitive, emotional, and technology-use factors to corresponding design requirements, including large fonts/high contrast, step-by-step flows, clear prompts, proactive and personalized responses, low-barrier onboarding, and family support.

Second, while multimodal interaction has been widely studied in HCI, its systematic adaptation for older-adult-oriented social robots remains underdeveloped. Many social robots for older adults still rely primarily on a single interaction modality (e.g., voice-only or touchscreen-dominant), and comparatively few studies present integrated multimodal solutions that combine channels such as voice, vision, and touch in a user-centered, context-sensitive manner. Even when multimodal approaches are adopted, technical integration often receives more attention than adaptation to age-related sensory, cognitive, and motor differences. For example, there remains limited research on how multimodal redundancy and complementarity can be tailored for older adults with hearing loss, vision decline, or varying levels of digital literacy. This indicates a need for frameworks that connect multimodal design principles with the specific functional characteristics and preferences of older users (Oviatt, 1999). Figure 1 summarizes this literature-based mapping by linking age-related physical, cognitive, emotional, and technology-use characteristics to corresponding multimodal interaction design requirements, and thereby provides a transition from the literature review to the framework development in the following section. Although this evidence comes from a different target population, systematic review and meta-analytic findings on social-robot interventions for children and young people on the autism spectrum also highlight heterogeneity in robot platforms, intervention settings, and outcome domains, as well as the need for rigorous evaluation before making efficacy claims (Kouroupa et al., 2022; Puglisi et al., 2022).

Third, although prior research has documented the potential of social robots for companionship and emotional support, there is still a theory-to-design gap in how emotion-related support processes are conceptualized and translated into interaction design and system architecture. Existing studies often discuss emotional support outcomes (e.g., loneliness reduction, engagement, comfort) at a descriptive level, but fewer studies explicitly map these outcomes to theoretically grounded mechanisms that can guide feature prioritization and evaluation planning. As argued in Section 2.2, an EI-informed lens may help bridge this gap by linking multimodal interaction channels to emotion perception, emotion understanding, and regulation-oriented support processes, while also maintaining a cautious distinction between design-oriented scaffolds and causal efficacy claims (Yen et al., 2024).

To address these gaps, the present study positions itself as an exploratory survey of user needs and preferences and a conceptual design-oriented investigation. Emotional intelligence is treated as a theoretical lens rather than a measured variable. The empirical part of the study, therefore, focuses on user needs, preferences, and perceived barriers, rather than on testing EI, emotional-health outcomes, or intervention effectiveness.

## 3. Multimodal Interaction Modalities for Social Robots in Later-Life Support

Guided by the EI-informed lens introduced in Section 2.2 and the literature-based requirement synthesis summarized in Figure 1, this section reviews six multimodal interaction channels and discusses their potential roles in later-life social-robot design. Rather than treating these modalities as validated intervention components, the section examines their usability, accessibility, privacy, and implementation constraints in relation to support-oriented design functions.

The significance of multimodal human–robot interaction in later-life social robots extends beyond merely integrating sensors and communication channels. It lies in establishing understandable, controllable, and sustainable interaction pathways within the sensory, cognitive, and contextual constraints of middle-aged and older users. These pathways can support daily task execution and social connection, thereby informing systems intended to provide perceived emotional support, and the design of systems intended to support these activities and perceived emotional support. Multiple existing studies indicate that channels such as voice, vision, gestures, and emotion each possess distinct advantages and boundary conditions in home and community settings (Oviatt, 1999). Their usability depends on the combined effects of contextual factors (Li et al., 2022) (noise, lighting, spatial constraints), individual capabilities (Glyde et al., 2011) (hearing, vision, and fine motor skills), and psychosocial factors such as privacy and trust (Zafrani et al., 2023). Therefore, this section systematically reviews the adaptability and limitations of different channels, drawing on the existing literature, and provides a comprehensive assessment of multimodal human–robot interaction applications for social robots in later-life support.

### 3.1. Visual Interaction

Visual interaction provides clear and verifiable feedback for navigation guidance, scenario demonstrations, and remote collaboration through interface presentation and environmental perception. As a core module of multimodal human–computer interaction, it also reduces the demands on working memory for multi-step tasks. First, the sensing layer captures facial expressions, gaze direction, and motion trajectories using RGB, RGB-D, depth, and infrared sensors. Next, the algorithm layer performs detection, tracking, feature extraction, and intent/emotion classification. Finally, the interface layer presents results and error prompts through text, icons, animations, or step-by-step visual guidance (Agravante et al., 2017; Picard, 2000). In social robots for later-life support, commercial models such as Pepper integrate RGB-D cameras and facial expression recognition modules to detect emotion-related cues perception among older users. This enables adaptive adjustments to dialogue strategies and prompt intensity, thereby enhancing the interaction experience (Amabili et al., 2023). In EI-informed terms, the visual channel primarily supports emotion perception and context interpretation, which can inform adaptive dialogue timing and response style. Overall, the visual channel offers intuitive information delivery and visible feedback, which benefits users with sufficient visual ability. However, in scenarios involving complex lighting, cross-cultural expression differences, or extremely subtle expressions, misjudgments are prone to occur, placing higher demands on algorithm robustness and cross-cultural adaptability. To reduce errors and user burden, the interface layer emphasizes large font sizes and high contrast, step-by-step visual guidance, and visible data collection status indicators. The data processing layer prioritizes on-device feature extraction while minimizing reliance on raw video footage, striking a balance between accessibility and privacy.

### 3.2. Voice Interaction

Voice interaction centers on Automatic Speech Recognition (ASR) and Natural Language Understanding (NLU), converting spoken commands or conversations into system intents and providing feedback via synthesized speech or brief text. First, the capture end performs speech activity detection and noise reduction; next, ASR transcribes the speech, with NLU/dialogue management analyzing intents and slots; finally, the system executes tasks and confirms or prompts with Text-to-Speech (TTS) synthesis combined with text and icons. In practical applications, voice interaction remains the most used foundational channel in home and community settings due to its low learning curve and high user affinity. From an EI-informed design perspective, voice interaction is also a primary channel for capturing emotional expression and supporting empathic or context-sensitive conversational responses (supporting emotion perception and understanding). It covers high-frequency tasks like daily conversation reminders and semantic searches, as illustrated by conversational agents and humanoid social-robot platforms such as Pepper (C.-Y. Chen et al., 2018; Lala et al., 2019; Rao et al., 2018). However, under real-world conditions involving regional dialect variation (particularly salient in China), environmental noise, and age-related hearing impairment, voice-only interaction pipelines are more vulnerable to recognition breakdowns and communication errors. A robust approach, therefore, involves combining voice with visual confirmation or gesture touch as redundant elements: when noise levels rise or recognition confidence drops, the system proactively suggests switching to visible large buttons or simple gestures. Confirmation and error correction can then be completed through the secondary channel. Upon successful recognition, secondary confirmation is provided via brief text and icons to reduce the subjective feeling of being “misunderstood.” At the conversational level, a collection of concise synonymous expressions should be compiled to reduce the burden of expression. Regarding privacy, non-continuous listening, prominent activation prompts, and one-click record clearing should be considered to support transparency, trust, and acceptance.

### 3.3. Affective Interaction

The core of affective interaction, in an EI-informed framework, lies in sensing, interpreting, and responsively supporting users’ emotion-related states through multimodal signals (e.g., voice, facial expressions, and posture), so that interaction strategies can be adapted in a socially and emotionally appropriate manner. First, multimodal fusion estimates emotional features, such as acoustic signals, facial Action Units (AUs)—standardized facial muscle movements—and postural rhythm. Second, a state machine/policy network adjusts prompt intensity, conversational style, and interaction intervals based on these estimates. Ultimately, the system conveys emotional feedback through both verbal and nonverbal cues. Related reviews indicate that appropriate emotional responses may enhance perceived understanding and support sustained engagement in some contexts (e.g., Pepper robot) (Del Angel et al., 2016). Affective interaction may support perceived responsiveness, social presence, and emotionally supportive experiences. However, current emotional recognition accuracy remains constrained by the complexity of multimodal signal processing, the diversity of training data, and hardware limitations. Additionally, data privacy and ethical concerns have drawn significant academic attention. Furthermore, the authenticity of robotic emotional feedback is limited, making long-term acceptance and trust uncertain for some users. In this study, affective interaction is treated as a design-oriented support mechanism rather than a validated therapeutic intervention component.

### 3.4. Somatosensory Interaction

Somatosensory Interaction (such as gesture recognition) utilizes cameras and sensors to perceive users’ limb movements, thereby enabling natural, contactless human–computer communication (H. Liu & Wang, 2018). First, it performs keypoint detection and trajectory tracking of hands/body; second, it classifies gestures based on spatiotemporal features and maps them to command sets; finally, it completes closed-loop confirmation and error correction through auditory/visual feedback. In social robots for later-life support, this interaction method provides an accessible entry point for older users with mobility limitations or speech impairments, commonly seen in rehabilitation training and entertainment interaction scenarios. For instance, rehabilitation robots and AI rock-paper-scissors robots analyze hand trajectories and movement amplitude to effectively support the personalized needs of users with motor impairments (Segal et al., 2020). However, recognition accuracy in gesture-based interaction is susceptible to environmental lighting, background clutter, and individual user variations. Factors such as hand tremors and reduced movement amplitude in older users may further increase the risk of misrecognition, demanding higher algorithmic fault tolerance and sensor precision (Feng et al., 2018). Therefore, when adopting somatosensory interaction, gesture sets should initially emphasize a small number of stable and easily repeatable actions. Retain a limited set of highly replicable fundamental movements, allow reasonable tolerances for amplitude and temporal sequencing, and provide comprehensible example prompts during the initial usage phase. When consecutive recognition failures occur, promptly switch to alternative channels to avoid adding cognitive load within an error loop. In this sense, the value of the somatosensory interaction channel lies in enhancing the system’s sustained interaction capability within complex scenarios. Although primarily used for accessibility and command input, somatosensory interaction may also support embodied reassurance and engagement in specific scenarios (e.g., rehabilitation or guided activities).

### 3.5. Electromyography Interaction

Surface electromyography (sEMG) serves as the foundation for electromyography-based interaction. By deploying sensors on the user’s skin surface to capture muscle electrical activity, combined with signal processing and pattern recognition, it enables intentional control of robots. The process involves: (1) signal acquisition and preprocessing (filtering, normalization); (2) feature extraction and intent recognition (traditional features/deep representations); and (3) intentions mapping to high-priority commands such as “confirm,” “help,” or “stop” with immediate feedback (Rusydi et al., 2019). Its advantage lies in its high sensitivity to subtle muscular intentions, enabling critical operations such as “confirm,” “help,” and “stop” without vocalization or large movements, thereby maintaining continuous usability under adverse conditions. Research prototypes of sEMG-controlled assistive robots and exoskeletons have shown the potential of muscle-signal-based control for users with severe motor impairments (Y. Liu et al., 2021). However, sEMG stability is susceptible to electrode adhesion, skin conditions (e.g., sweat, oil), posture changes, and fatigue; prolonged wear may also cause discomfort and increased maintenance burden. Therefore, while considering electromyographic interaction, one should prioritize a limited set of high-value commands for infrequent use in hazardous situations or special contexts. Integration with other sensing channels is recommended to prevent false triggers. At the interaction level, establishing complementary pathways with visual and voice systems is advised, enabling automatic switching when signal quality deteriorates. Furthermore, during data processing, both usability and user privacy must be balanced, retaining only task-essential feature values. In this framework, sEMG is primarily positioned as an accessibility and continuity channel rather than as a primary modality for affective interaction.

### 3.6. Brain–Computer Interface (BCI) Interaction

Brain–computer interfaces convert users’ mental selections into control commands by capturing neural signals such as electroencephalograms (EEG) and decoding their features, addressing extreme accessibility needs for individuals with severe limitations in movement and speech (Thomas et al., 2013). The process involves: first, acquiring EEG signals and suppressing artifacts (eye movements/muscle activity); second, constructing features and classifying intentions (using paradigms like P300/SSVEP/imagined movement); finally, commands are mapped to a limited set of high-value instructions and confirmed via multi-channel feedback. In research and development contexts, commercial wireless EEG headsets such as the Emotiv EPOC X demonstrate the feasibility of portable EEG acquisition for BCI-related studies. However, their relevance to later-life social-robot interaction remains exploratory and constrained by signal noise, calibration burden, user comfort, cognitive load, and individual variability. Clinical closed-loop neurostimulation systems such as the NeuroPace RNS System demonstrate that neural signals can be monitored and used for responsive medical intervention in specific neurological conditions, but such systems are medical devices rather than social-robot interaction interfaces and should not be directly equated with everyday BCI-based robot control (Chakravorti et al., 2019). Therefore, in later-life social-robot contexts, BCI should be positioned as a high-constraint accessibility channel for essential commands rather than as a routine channel for socio-emotional interaction. Brain–computer interaction should be configured to support only a limited set of high-value commands, such as emergency confirmation or stop commands, with multi-channel confirmation and safeguards against false triggers. User privacy must also be considered, and feature extraction should, where feasible, minimize the retention of raw neural data.

### 3.7. Summary

**Figure 2:**
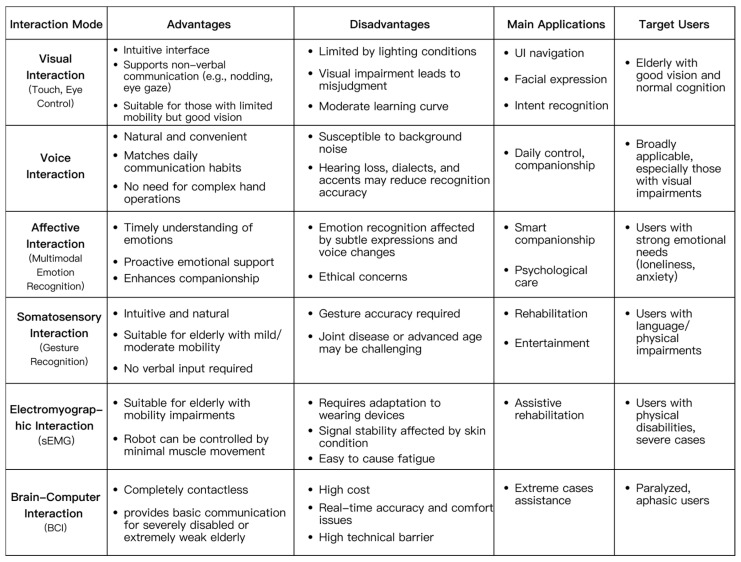
Comparative matrix of interaction modes for social robots for later-life support. This matrix summarizes six modalities—visual, voice, affective, somatosensory, sEMG, and BCI—in terms of advantages, limitations, main application scenarios, and target user conditions to guide conceptual modality selection and system design.

Based on the above content, this paper analyzes and summarizes the comparative advantages and disadvantages of different interaction methods for social robots for later-life support. The results are shown in Figure 2.

In summary, different interaction methods each have their own scope of application and distinct advantages in the field of social robots for later-life support, but they all have significant limitations. From a practical application perspective, voice interaction remains the most widely adopted and practically foundational channel in many home and community scenarios. For many older users, voice communication inherently offers intuitive and approachable interaction. It not only fulfills daily operational needs but also supports emotional companionship and remote care. In many cases, voice interaction alone can support effective task-oriented interaction and basic companionship, although its reliability may be constrained by hearing impairment, dialectal variation, and environmental noise.

However, voice interaction alone cannot fully address the complex needs of all older users. For some older users, factors such as hearing impairments, cognitive decline, or regional accents may affect speech recognition accuracy. While modalities like vision, motion sensing, and emotion detection each have limitations, they can serve as valuable complements in specific scenarios. For instance, though visual interaction is significantly influenced by eyesight and environmental conditions, it still offers unique value in interface navigation, facial expression recognition, and spatial guidance. Emotion recognition also functions not as an independent input channel but as a valuable assistant to the aforementioned modalities. It enhances the companionship, warmth, and appeal of social robots for older users by adjusting tone and frequency. Gesture-based interaction provides simplified operation channels for users with mobility or speech limitations, expanding interaction options. While electromyography (EMG) and brain–computer interface (BCI) interactions remain niche, they may be meaningful for users with severe motor or speech limitations who cannot easily use conventional channels. In specific rehabilitation, assistive, or silent communication scenarios, these modalities serve as the last means for users to engage with the world, potentially supporting independence and social participation in constrained contexts. Therefore, the analysis suggests a combined-modality strategy in which voice can serve as the primary channel for routine interaction, supported by visual confirmation and selected gesture-based backup. Affective interaction can serve as an enrichment layer for social and emotional responsiveness, while sEMG and BCI should be positioned as high-value auxiliary channels in specialized contexts, particularly when conventional interaction methods are infeasible. This configuration aims to balance usability, accessibility, continuity, and privacy in social robots for later-life support.

The comparative analysis and modality-combination strategy presented in this section are conceptual and design-oriented, and are intended to support framework development and future evaluation planning rather than to establish causal effects on emotional-health outcomes in the present study.

## 4. Questionnaire Survey and Descriptive Findings

### 4.1. Questionnaire Design, Recruitment, and Data Collection

To explore user needs, preferences, and perceived barriers related to multimodal interaction with later-life social robots, this study designed and implemented a structured questionnaire. In line with the EI-informed lens developed in Section 2.2, the questionnaire was intended to provide demand- and constraint-side evidence for multimodal and affective interaction design, rather than direct evidence of the efficacy of emotional-health interventions.

The questionnaire items were developed through a literature review and conceptual analysis of multimodal interaction and emotional-support processes. The items assessed recognition, willingness to use, perceived barriers, and functional requirements across six interaction modalities using Likert-scale and multiple-choice formats. The questionnaire was pilot tested with three middle-aged volunteers to improve clarity and face validity. Detailed questionnaire content is provided in the Appendix A.

Participants were recruited through convenience sampling in urban and suburban community centers, senior universities, and online platforms between July and August 2025. Adults aged 45 and above were invited to participate. Data were collected through both online and offline paper-based questionnaires. A total of 203 responses were collected, of which 199 were valid, resulting in a validity rate of 98.0%. Prior to completing the questionnaire, all respondents received an informed-consent statement explaining the study’s purpose, the voluntary nature of participation, the anonymity of responses, and data confidentiality. No personally identifiable information was collected, and the study was conducted as minimal-risk social research.

Therefore, the present survey dataset does not permit causal inference about the emotional-health outcomes or intervention effectiveness.

### 4.2. Sample Population Information

**Figure 3:**
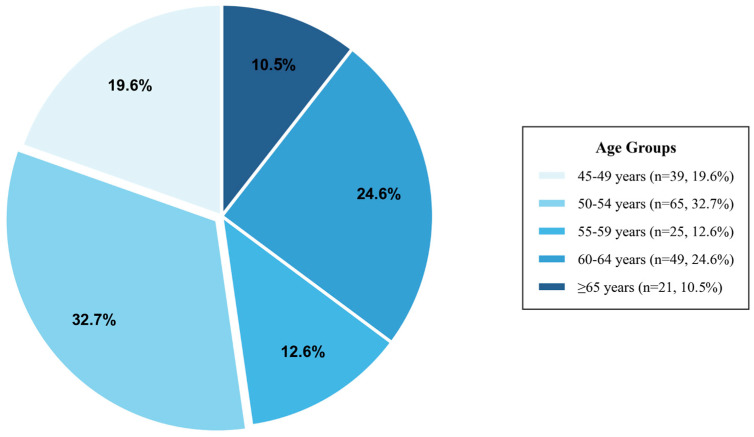
Age Distribution of Study Participants (*N* = 199). The sample was predominantly middle-aged (45–64 years: 89.4%), with a smaller proportion aged 65 years and above (10.6%).

The sample data were preprocessed, and descriptive statistical analyses were performed, yielding Table 1. Among the 199 valid responses collected, the gender distribution showed 86 males (43.2%) and 113 females (56.8%), with females comprising a somewhat larger proportion. Importantly, the age distribution (Figure 3) reveals that our sample primarily represents middle-aged adults who are prospective elderly service users: 32.7% (65 individuals) were aged 50–54, 24.6% (49 individuals) aged 60–64, 19.6% (39 individuals) aged 45–49, 12.6% (25 individuals) aged 55–59, with only 10.6% (21 individuals) aged 65 and above. Although this sample cannot fully represent the broader older-adult population, it is informative for identifying anticipatory needs and acceptance patterns among prospective later-life users.

Regarding educational attainment, the sample predominantly held secondary qualifications: high school/vocational school accounted for 32.6% (65 individuals), junior high school for 19.0% (38 individuals), and college/technical school for 14.5% (29 individuals); primary school and below constituted 13.5% (27 individuals), bachelor’s degree for 18.6% (37 individuals), and master’s degree or higher for 4.5% (9 individuals), indicating a relatively limited proportion of higher education.

Marital and residential patterns reflect relatively stable family structures. Among respondents, 188 (94.4%) were married, 7 (3.5%) were divorced, and 4 (2.0%) were widowed. The most common residential arrangement was living with a spouse (91.9%, 183 individuals), followed by living with children (6.0%, 12 individuals); living with relatives or friends accounted for 1.5% (3 individuals), while living alone was relatively low (0.5%, 1 individual). Overall, this sample primarily consists of middle-aged and older married respondents residing in urban and suburban areas, with women slightly outnumbering men. They possess a family support network centered around their spouses. This demographic structure aligns with the family-community context examined in this paper.

### 4.3. Data Analysis and Results

Descriptive statistics, including percentages and cross-tabulations, were used to examine patterns of awareness, willingness to use, perceived barriers, and modality–function co-selection. Because the same respondents evaluated multiple interaction modalities, the present analysis does not make inferential comparisons across modalities. The results are therefore interpreted as exploratory patterns that inform design implications rather than as statistically validated differences or causal evidence.

#### 4.3.1. Analysis of User Awareness, Usage Willingness, and Perceived Barriers Across Interaction Modalities

**Figure 4:**
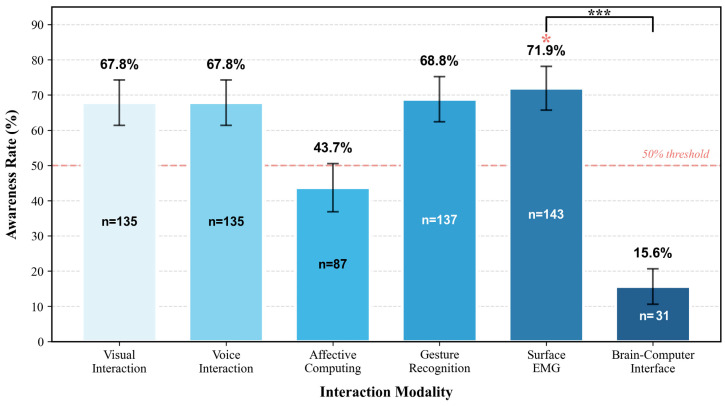
Reported Awareness of Multimodal Interaction Modalities (*N* = 199). Error bars indicate 95% confidence intervals based on Wilson score intervals. The reported sEMG awareness rate may include confusion with clinical electromyography or other physiological sensing technologies. Results are interpreted descriptively. Note: The red asterisk denotes the note regarding possible confusion with clinical electromyography, whereas the black triple asterisks indicate a statistically significant difference between the connected modalities (χ^2^ test, *p* < 0.001).

**Figure 5:**
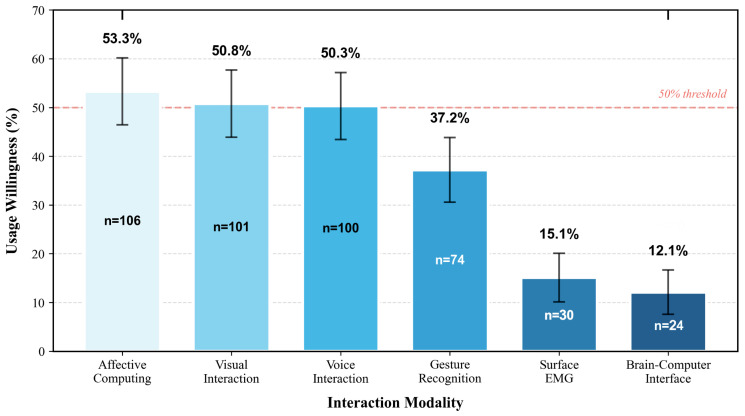
Reported Usage Willingness Across Multimodal Interaction Modalities (*N* = 199). Error bars indicate 95% confidence intervals based on Wilson score intervals. These results are interpreted descriptively rather than as inferential comparisons across modalities.

**Figure 6:**
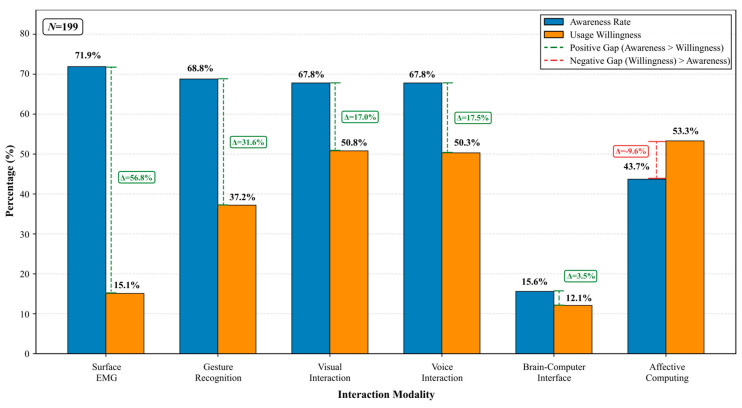
Awareness-Willingness Gap in Multimodal Interaction Technologies (*N* = 199). Blue bars indicate reported awareness rates, and orange bars indicate reported willingness to use. Positive gaps indicate awareness exceeding willingness, while negative gaps indicate willingness exceeding awareness. The gap values are descriptive indicators of awareness–willingness mismatch rather than inferential test results.

Survey data reveal clear disparities between respondents’ awareness and willingness to use across different interaction modalities (Figure 4, Figure 5 and Figure 6). As shown in Figure 4, surface electromyography (sEMG) exhibited the highest reported awareness rate (71.9%, *n* = 143), followed by gesture recognition (68.8%, *n* = 137) and both visual and voice interactions (each 67.8%, *n* = 135). In contrast, affective computing showed a moderate awareness rate (43.7%, *n* = 87), while brain–computer interface (BCI) interaction had the lowest awareness (15.6%, *n* = 31). The relatively high reported awareness of sEMG should be interpreted cautiously, as some respondents may have associated it with more familiar clinical electromyography procedures rather than sEMG as a human–robot interaction modality. Future surveys should include clearer descriptions and validation items to better distinguish conceptual familiarity from an accurate understanding of technology.

Regarding willingness to use (Figure 5), respondents showed the highest willingness for affective computing (53.3%, *n* = 106), followed by visual interaction (50.8%, *n* = 101) and voice interaction (50.3%, *n* = 100). Gesture recognition received moderate willingness (37.2%, *n* = 74), whereas sEMG (15.1%, *n* = 30) and BCI (12.1%, *n* = 24) showed comparatively low willingness to use. These results suggest that participants generally favored interaction modalities that are perceived as intuitive, familiar, or emotionally meaningful, while remaining more cautious toward high-barrier or device-dependent modalities.

As illustrated in Figure 6, the awareness-willingness gap analysis further highlights differences between familiarity and adoption intention across modalities. sEMG showed the largest positive gap (Δ = +56.8%), indicating high reported awareness but very low willingness to use, which may reflect concerns regarding practicality, wearability, maintenance burden, or uncertainty about real-world usefulness. Gesture recognition also showed a substantial positive gap (Δ = +31.6%), suggesting that although respondents are aware of gesture-based interaction, they may perceive its operation as less convenient or reliable in daily use. Visual interaction (Δ = +17.0%) and voice interaction (Δ = +17.5%) also showed positive gaps, indicating that familiarity does not necessarily translate into strong adoption intention, possibly due to concerns about lighting conditions, privacy, noise, dialect variation, hearing limitations, or recognition accuracy in real-world contexts. By contrast, affective computing was the only modality showing a negative gap (Δ = −9.6%), meaning usage willingness exceeded awareness. This pattern suggests latent demand for emotionally responsive interaction, even among respondents who may not be familiar with the technical term.

From an EI-informed design perspective, the relatively high willingness to use affective interaction may indicate user interest in emotion-related cue perception, empathic responsiveness, and low-risk regulation-oriented support. However, this finding reflects perceived desirability and acceptability, not demonstrated effectiveness in improving emotional-health outcomes.

Regarding perceived barriers and qualitative feedback, respondents reported that voice interaction may be affected by dialects, accents, environmental noise, and hearing limitations, which can reduce reliability in practical use. Visual interaction may be constrained by age-related declines in vision and lighting conditions. Newer modalities such as gesture recognition, sEMG, and BCI face additional barriers, including device wear burden, unfamiliar operating procedures, and higher learning costs. Some respondents also expressed interest in affective interaction while voicing concerns that robots might fail to accurately interpret emotions, thereby producing interactions that feel unnatural or overly mechanical. These findings suggest that while respondents generally maintained an open attitude toward multimodal interaction, actual acceptance remains strongly shaped by cognitive habits, health conditions, and prior technology experience. Therefore, multimodal redundancy, personalized adaptation, and transparent interaction design remain important directions for future system optimization.

Some questionnaire items may have been interpreted differently by respondents; therefore, the results should be understood as exploratory indicators of perceived awareness and willingness rather than conclusive evidence of stable preferences.

#### 4.3.2. User Functional Requirements Analysis

Building on the analysis of respondents’ awareness and attitudes toward multimodal interaction systems, this section further examines their functional priorities for social robots designed for later-life support. In the context of this study, the selected function items are interpreted as service needs and application-layer design inputs that can inform subsequent conceptual framework development.

**Figure 7:**
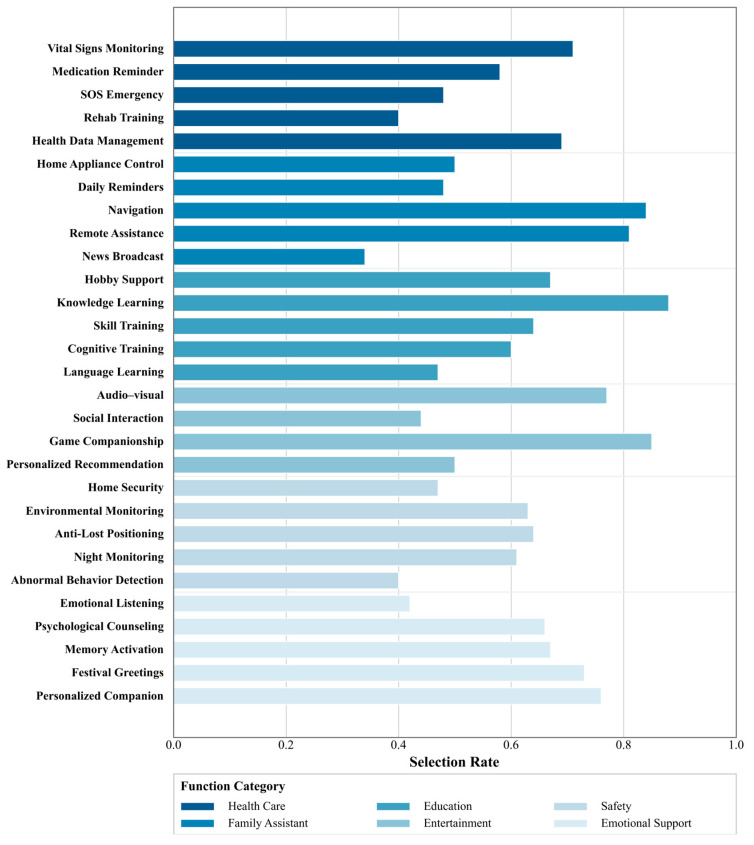
Selection Rate of Function Items Grouped by Category (*N* = 199). Bar length indicates the percentage of respondents selecting each function item. Colors denote the six functional categories.

**Figure 8:**
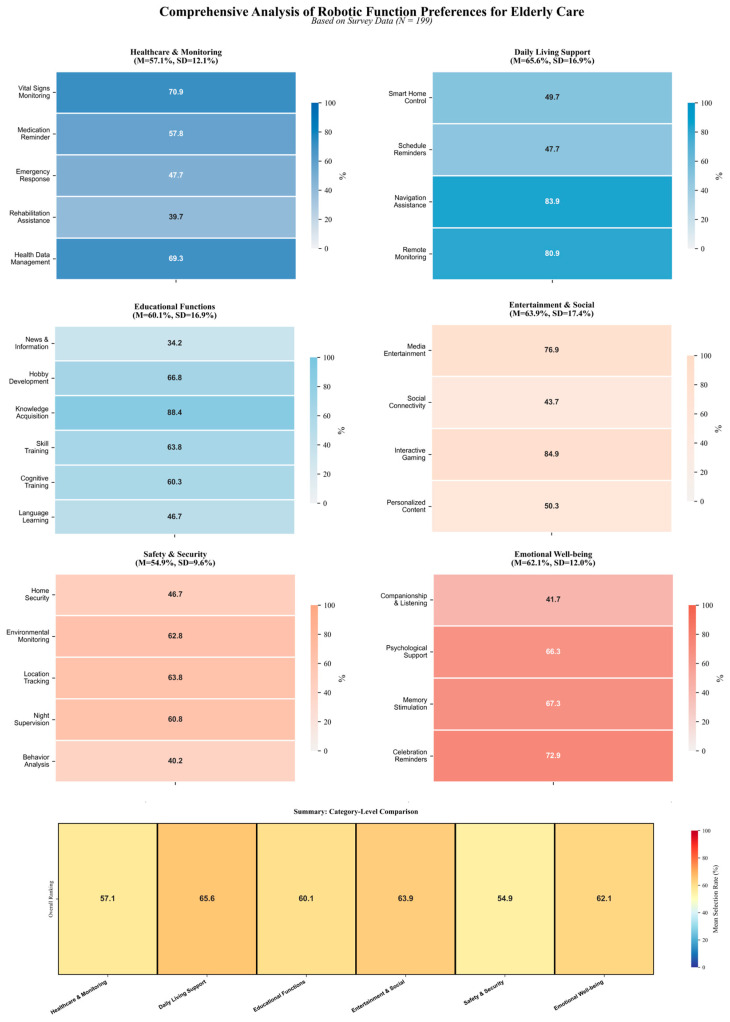
Function Preferences for Later-Life Social-Robot Support Across Six Functional Categories (*N* = 199). Heat maps show selection rates for function items across six categories. Values indicate the percentage of respondents selecting each item. Category-level means are shown descriptively.

Based on the survey data, respondents showed diverse but clearly structured preferences across function items (Figure 7 and Figure 8). At the individual-item level, knowledge learning (88.4%), personalized companionship (85.4%), game companionship (84.9%), navigation guidance (83.9%), and remote assistance (80.9%) were among the most frequently selected functions. These were followed by items such as audio-visual entertainment (76.9%), festival greetings (72.9%), vital signs monitoring (70.9%), health data management (69.3%), and psychological counseling (66.3%), suggesting strong demand for a combination of practical support, sustained engagement, and emotional-health-related assistance.

At the category level (Figure 8), higher overall selection rates were observed for daily living support, entertainment and social interaction, emotional well-being support, and healthcare/monitoring functions. This pattern indicates that respondents value social robots not only as assistive tools for safety and routine tasks but also as companions that support everyday engagement, emotional connection, and quality of life. Supportive functions such as medication reminders (57.8%), night monitoring (60.8%), cognitive training (60.3%), and environmental monitoring (62.8%) also received substantial support, indicating that safety and capability-maintenance needs remain important alongside companionship and entertainment.

By contrast, some functions showed relatively lower selection rates (e.g., emergency/SOS-related items, abnormal behavior detection, and emotional listening). These lower rates should not be interpreted as indicating low importance in and of themselves. Rather, they may reflect scenario specificity (e.g., emergency functions being perceived as low-frequency), uncertainty about practical usefulness, trust and privacy concerns, or ambiguity in respondents’ interpretation of certain function labels. This suggests that future systems should prioritize clearer feature communication, configurable function bundles, and context-sensitive deployment strategies rather than assuming uniform demand across all users.

From an EI-informed design perspective, the relatively high willingness to use affective interaction may indicate user interest in emotion-related cue perception, empathic responsiveness, and low-risk regulation-oriented support. However, this finding reflects perceived desirability and acceptability, not demonstrated effectiveness in improving emotional-health outcomes. Items within the Emotional Support/Emotional Well-being category can therefore be interpreted as potential user-facing support components rather than validated intervention components. For example, emotional listening may support perceived social presence and empathic responsiveness; psychological support, understood here as lightweight guidance or prompts rather than clinical therapy, may support regulation-oriented assistance; memory activation may support reminiscence and positive affect; and personalized companionship may support relational continuity and sustained engagement. In this sense, the function-preference results provide design-oriented input for identifying potential emotional-support components to be considered in future prototype development, rather than evidence that these components produce emotional-health outcomes.

Overall, the findings suggest that social robots for later-life support should combine practical assistance, engagement and learning support, safety-related monitoring, and socially and emotionally supportive interaction in a configurable manner. In the present study, these functional preferences are used as design-oriented evidence for framework construction and component prioritization, rather than as evidence of intervention effectiveness.

#### 4.3.3. Correspondence Between Different Interaction Methods and Required Functions

**Figure 9:**
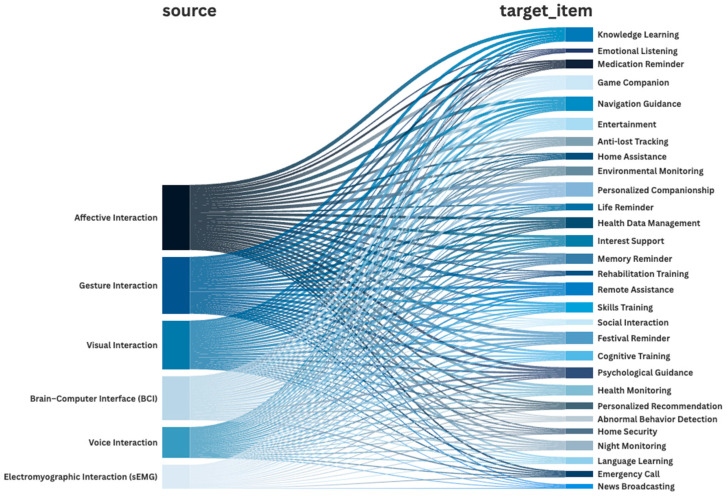
Co-Selection Mapping Between Interaction Modalities and Function Items (*N* = 199). The left side shows interaction modalities, while the right side shows function items. Thicker lines indicate a higher number of respondents who selected both the interaction modality and the function item. Colors are used solely to distinguish lines and do not represent statistical differences.

The correspondence shown in Figure 9 reflects co-selection patterns between interaction modalities and function items in the questionnaire and is used here as a design-oriented mapping cue. It does not, by itself, establish causal effectiveness or optimal modality–function assignments.

Overall, connections predominantly cluster around high-priority functions such as “knowledge learning, gaming companionship, navigation guidance, and personalized companionship,” suggesting that these functions were frequently co-selected alongside multiple interaction methods. This suggests that these functions were frequently associated with multiple interaction modalities in respondents’ preferences. Affective, gesture-based, and visual interaction showed relatively broad co-selection coverage across function items.

In learning and entertainment-related categories, visual and affective interactions were frequently co-selected, possibly reflecting respondents’ preference for understandable feedback and emotionally engaging interactions. For daily assistance functions, voice and visual interaction were common combinations, with gesture interaction serving as a potential fallback when speech is constrained by noise, accents, or hearing limitations. Health-related functions were more frequently associated with visual and voice feedback, while safety and monitoring functions appeared more scenario-specific. sEMG and BCI showed fewer co-selection links, which is consistent with their conceptual positioning as low-frequency, high-value backup channels within the proposed framework.

## 5. Conceptual Architecture for Multimodal Social Robot in Later-Life Support

### 5.1. Conceptual Framework Construction

**Figure 10:**
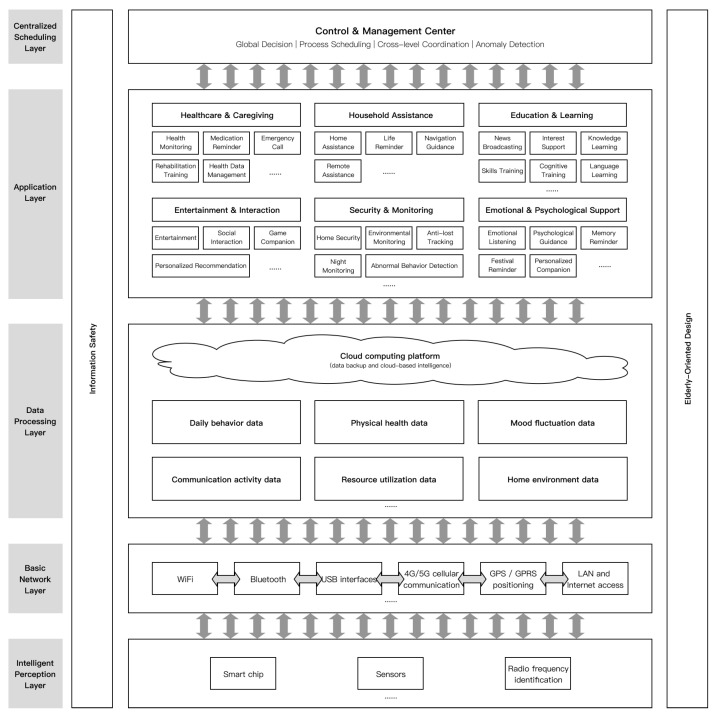
Conceptual Five-Layer Architecture for Multimodal Social Robots in Later-Life Support. Note: A five-layer conceptual framework for elderly-oriented multimodal interaction systems. This diagram is intended as a descriptive scaffold to support comparative analysis and future integration studies, rather than as a prescriptive technical blueprint. Arrows indicate information flow and interaction between adjacent layers.

In this study, the five-layer architecture is proposed as a conceptual and design-oriented scaffold for organizing multimodal interaction channels, service functions, and age-friendly safeguards in later-life social-robot design. Building on the preceding literature review and the questionnaire-based findings in Section 4, we define multimodal interactive social robots for the older users as shown in Figure 10. The system architecture comprises five primary layers, arranged from bottom to top as follows: Intelligent Perception Layer, Basic Network Layer, Data Processing Layer, Application Layer, and Control & Management Center. Information security and age-friendly design are treated as cross-layer safeguards rather than as isolated modules.

1. **Intelligent Perception Layer:** The underlying layer is the Intelligent Perception Layer, responsible for real-time collection of multi-source information, including voice, images, motion, surface electromyography (sEMG), and electroencephalography (EEG). It supports the perception of diverse physiological and behavioral, and contextual cues from later-life users. The system gathers user status and environmental cues through microphones, cameras, depth sensors, IMU/touch, RFID, and optional sEMG/EEG devices. It performs local noise reduction, clock synchronization, and feature extraction (e.g., speech transcription and prosody statistics, pose/gesture key points, gaze vectors, scene events, low-dimensional sEMG/EEG features), where feasible, reducing or discarding raw data after feature extraction. This layer outputs structured events and features with temporal details. All sensors can be configured with visual indicators and dual hardware/software switches to prevent privacy violations and erroneous data collection. For age-friendly design, sensor placement, interaction prompts, and interface design should adhere to the principles of “large font size, high contrast, distributed guidance, and motion/timing tolerance.” During the initial user setup, provide a lightweight “demonstration-follow-feedback” tutorial to reduce the learning curve. Security measures include firmware integrity verification and local key protection to prevent device tampering.
2. **Transmission Network Layer:** Collected data is transmitted reliably and efficiently to the upper layers via the foundational network layer, using multiple communication methods such as WiFi, Bluetooth, and 4G/5G to ensure data integrity and real-time delivery. Transmission Priority Differentiation by Business Criticality: Events such as emergency calls or abnormal falls should use high-priority channels, while routine interactions and daily activities should use low-priority channels. Link status (latency, packet loss, jitter) is continuously monitored for automatic rerouting and retransmission. In offline or high-latency scenarios, the network layer triggers local fallback: essential interactions and alerts remain functional, with synchronization occurring upon network restoration. All transmissions employ end-to-end encryption and mutual authentication. Critical control messages are signed and protected against replay attacks. Remote assistance and audio/video calls utilize point-to-point encryption with minimized metadata collection. For elderly users, this layer ensures that core services remain uninterrupted, regardless of network quality, with clear, understandable status indicators (e.g., network status alerts, offline notifications, and automatic retries).
3. **Data Processing Layer:** The Data Processing Layer uses edge/cloud computing resources to process structured events, validate data quality, support multimodal inference, and coordinate service responses. It completes a closed-loop process encompassing streaming ingestion, quality validation, event bus, model services, and feature/log storage, while also handling multimodal collaboration and inference. Data domains align with the diagram, covering six categories: daily behavior, physical health, emotional fluctuations, communication activities, resource utilization, and home environment. Each domain is aligned with session IDs and unified timestamps to facilitate cross-modal fusion. Adhering to “edge-first, minimal collection” principles, the cloud retains only de-identified features and aggregated statistics, with defined retention periods and automated deletion policies. The collaborative strategy implemented at this layer is as follows: (1) Voice serves as the primary channel for routine interactions, (2) Vision is used for explicit confirmation and multi-step guidance, (3) Gestures/touch act as fallbacks during noise interference or reduced confidence, (4) Emotional cues function as “gentle amplifiers” for subtle adjustments to tone or reminder pacing, (5) sEMG/BCI mappings handle low-frequency, high-value commands (confirm/stop/help). From an EI-informed perspective, this layer operationalizes multimodal coordination by linking channels to emotion-relevant support processes (e.g., affective cue sensing, empathic response adjustment, and regulation-oriented prompt pacing) under confidence and safety constraints. When multiple channels trigger simultaneously or conflict, the system maintains safety through explicit confirmation and short-term revocation. If recognition confidence persistently declines, the strategy automatically switches to more reliable channels. This layer also maintains anomaly detection (e.g., behavioral irregularities, emotion-related risk cues, abnormal resource consumption) and reports alerts to central control.
4. **Service Application Layer:** The Service Application Layer delivers modular services across six application clusters that align with the questionnaire-derived functional priorities and the framework shown in Figure 10: (1) Healthcare & Caregiving (e.g., health monitoring, medication reminders, rehabilitation training, health data management, emergency calls), (2) Household Assistance (e.g., home assistance, daily reminders, navigation guidance, remote assistance), (3) Education & Learning (e.g., news broadcasting, interest support, knowledge learning, skills training, cognitive training, language learning), (4) Entertainment & Interaction (e.g., entertainment, social interaction, game companionship, personalized recommendations), (5) Security & Monitoring (e.g., home security, environmental monitoring, anti-lost tracking/wandering prevention, night monitoring, abnormal behavior detection), and (6) Emotional & Psychological Support (e.g., emotional listening, psychological support prompts, memory reminders/activation, festival greetings, personalized companionship).
  Within an EI-informed interpretation, the Emotional & Psychological Support cluster includes user-facing design-oriented components such as empathic listening, regulation-oriented support prompts, reminiscence/memory activation, and relational companionship cues. These modules are intended to support emotional-health-related interaction needs in everyday contexts, rather than to serve as standalone clinical interventions. All critical operations in these service modules should follow a double-confirmation protocol, with user-cancel options and configurable caregiver/family authorization for remote collaboration and assistance.
5. **Central Control Layer:** The Central Control Layer is responsible for global decision-making, process scheduling, cross-layer coordination, and anomaly handling. Its core components are the strategy engine and personalized configuration: based on environmental signals such as noise, lighting, and network quality, combined with profiles of elderly users’ hearing, vision, and motor abilities, and by analyzing past user interactions, it dynamically allocates channel weights and default communication paths to balance the efficiency and reliability of multimodal interactions. When events such as suspected falls, prolonged inactivity, device malfunctions, or privacy risks occur, tiered alerts and emergency protocols are triggered: local notifications are issued first, followed by contact with family members or caregivers. In critical situations, emergency assistance hotlines are directly dialed.

Information security and age-friendly design are integrated across all layers. The framework incorporates mechanisms for data encryption, privacy protection, and permission management, together with age-friendly interface and interaction design principles intended to improve usability, transparency, and trust for older adults.

Through this multi-layer, end-to-end architecture, the framework supports a closed-loop flow spanning data collection, transmission, multimodal analysis, and modular service delivery. In the present study, this architecture is positioned as a design-oriented and implementation-facing scaffold for integrating multimodal interaction and EI-informed support functions in older-adult-oriented social robots, rather than as a validated proof of intervention effectiveness.

### 5.2. Conceptual Mapping of Representative Social Robots

Based on the literature review and publicly available product documentation, six representative social-robot platforms were selected for conceptual mapping: Paro (*Paro—ROBOTS: Your guide to the world of robotics*, n.d.), Pepper (*Pepper (robot)—Wikipedia*, n.d.), ElliQ (*ElliQ—Atlas of the future*, n.d.), LOVOT (*LOVOT—ROBOTS: Your guide to the world of robotics*, n.d.), Mabu (Winn, 2019), and NAO (Blavette et al., 2022). These platforms were selected because they represent different functional orientations in later-life support, including therapeutic companionship, humanoid social communication, proactive aging-in-place companionship, affective companionship, chronic-disease management, and robot-mediated health-promotion support.

The purpose of this mapping is not to provide empirical validation of the proposed architecture. Rather, it is used as a design-oriented illustration to illustrate how the five-layer framework can consistently describe heterogeneous social-robot systems. Accordingly, Table 2 summarizes each platform in terms of representative perception/input channels, data-processing focus, application-layer functions, and age-friendly design orientation.

1. Layered Correspondence and Abstraction Capacity. The mapping suggests that the proposed five-layer structure can provide a consistent descriptive scaffold for comparing heterogeneous social-robot systems.

**Perception & Network Layers:** The selected robots rely on different perception and interaction channels. For example, PARO emphasizes tactile and auditory interaction; LOVOT uses thermal sensing, depth sensing, and embodied presence cues; Pepper and NAO rely more strongly on humanoid embodiment, speech, gestures, and visual interaction; while ElliQ and Mabu emphasize conversational and screen-based interaction for home-based support. These examples show that the perception layer can accommodate diverse sensing inputs beyond standard audio-visual data.

**Data Processing to Decision Making:** The Data Processing Layer provides a way to describe different forms of interaction logic and user-state interpretation across platforms. For instance, ElliQ uses daily routine-related information to support proactive prompts, while Mabu focuses on symptom- and medication-related user reports for chronic-disease management. In this sense, the framework helps organize different data-processing focuses, but this mapping should not be interpreted as a technical validation of each platform’s internal algorithmic mechanisms.

2. Coverage of Application-Layer Functions. The Application Layer provides a structure for comparing the service orientations of different social robots.

**Healthcare & Caregiving:** Mabu illustrates how chronic-disease management, medication-related reminders, and daily health check-ins can be mapped to healthcare-oriented service modules.

**Emotional & Psychological Support:** PARO and LOVOT illustrate companion-oriented forms of emotional support and affective engagement. PARO represents therapeutic companionship through tactile and zoomorphic interaction, while LOVOT emphasizes bonding, warmth, eye contact, and social presence. These examples support the descriptive coverage of the Emotional and Psychological Support cluster, but they do not by themselves establish therapeutic effectiveness.

**Entertainment, Training, and Health-Promotion Support:** Pepper and NAO illustrate how humanoid robots can support social communication, exercise/training activities, and health-promotion education. For example, NAO has been examined in robot-mediated health-promotion activities among older adults in geriatric facilities. These cases are used as conceptual reference points rather than as evidence of formal framework validation.

3. Cross-Cutting Role of Design Pillars. The mapping also highlights the cross-cutting role of age-friendly design across different robot forms and service models.

**Elderly-Oriented Design:** The selected platforms adopt different strategies to reduce interaction burden and support acceptance. PARO uses a soft zoomorphic form and tactile interaction; ElliQ uses a non-humanoid companion form and proactive prompts; LOVOT emphasizes warmth and eye contact; Mabu uses personalized conversation for health-related routines; and Pepper and NAO use humanoid gestures and multimodal feedback. These differences suggest that age-friendly design should be treated as a cross-layer design principle rather than as a single interface feature.

The mapping results (summarized in Table 2) suggest that the proposed architecture can accommodate heterogeneous social-robot designs within a common descriptive framework. In particular, it provides a way to compare companion-oriented robots, humanoid communication robots, proactive home companions, and healthcare-oriented robots across perception channels, data processing logic, application-layer functions, and age-friendly design strategies. In the present study, this mapping is used to support conceptual clarification and design discussion, rather than to claim formal validation of technical performance, user acceptance, or intervention effectiveness.

## 6. Discussion: Limitations, Ethical Considerations, and Implementation Challenges

### 6.1. Limitations and Future Work

This study presents several limitations that should be acknowledged:

1. **Sample representativeness**. The sample was dominated by respondents aged 45–64, while participants aged 65 and above accounted for only 10.5%. Therefore, the findings should be interpreted primarily as the needs and preferences of prospective later-life users rather than as representative evidence for the broader older-adult population. Future studies should recruit a larger and more diverse sample of adults aged 65 and above and conduct field studies in home, community, and care settings.
2. **Intervention scope.** This study did not evaluate an emotional-health intervention or measure emotional intelligence as an empirical variable. Therefore, the proposed architecture should be understood as a conceptual design framework rather than a validated intervention model. Future work should implement prototypes and examine whether specific interaction components can support emotional well-being, companionship, usability, and sustained engagement.
3. **Measurement validity concern.** The study relied on self-reported questionnaire data. Measurement validity concern. The study relied on self-reported questionnaire data. The unexpectedly high awareness rate for sEMG (71.8%) may reflect confusion between sEMG and more familiar physiological sensing technologies or clinical electromyography. Future surveys should provide clearer definitions, examples, and comprehension checks for technical terms.
4. **Theoretical depth.** The analysis was primarily descriptive. Although this study refers to the Technology Acceptance Model and emotional intelligence as theoretical lenses, it did not operationalize these constructs or test causal pathways. Future research could combine validated scales, paired repeated-measures analyses, structural equation modeling, and longitudinal prototype evaluation to examine the relationships among user characteristics, modality preferences, acceptance, and emotional-support outcomes.

Despite these limitations, this study provides an exploratory basis for connecting user needs, multimodal interaction modalities, and conceptual system architecture in later-life social-robot design.

### 6.2. Ethical Considerations and Implementation Challenges in Multimodal Human–Robot Interaction for Later-Life Support

Multimodal interaction can improve accessibility and flexibility in social robots for later-life support, but it also introduces ethical and implementation challenges. Because such systems may collect voice, image, behavioral, physiological, and potentially neural data, privacy protection, transparency, user control, and accountability need to be considered across the full data lifecycle.

**Challenges in Multimodal Data Fusion and Robustness.** Multimodal data fusion can improve interaction reliability because different modalities provide complementary information under different conditions. However, it may also introduce redundancy, channel conflict, and error propagation. In real-world deployments, speech recognition may degrade in the presence of environmental noise, dialectal variation, or hearing limitations, while visual sensing may be affected by lighting, occlusion, and privacy concerns. These issues are particularly important for older users, for whom technical instability can quickly become usability and trust problems. Future work should therefore prioritize robust fusion strategies that support confidence-aware modality switching, explicit confirmation for safety-critical actions, and graceful fallback mechanisms when one or more channels become unreliable.

**Personalization and boundaries of affective interaction.** Affective interaction may increase perceived responsiveness and sustained engagement, but inaccurate emotion inferences or over-interpretation can create ethical risks such as discomfort, mistrust, or inappropriate system responses. In this study, affective interaction is therefore positioned as a low-risk assistive feature rather than as a diagnostic or clinical function. It may be used to adjust tone, pacing, reminder intensity, or light social prompts but should not be used to make medical or psychological judgments. Users and, where appropriate, caregivers should be able to enable, disable, and configure emotion-related features.

**Cultural adaptability, fairness, and age-related diversity.** Multimodal systems may perform unevenly when trained or evaluated on culturally narrow, linguistically limited, or age-unbalanced datasets. Facial expressions, gestures, dialects, speech patterns, and movement amplitude vary across users and contexts. These variations can affect recognition accuracy and interaction quality. Future systems should therefore include age-diverse and culturally diverse evaluation data, report subgroup performance where feasible, and support ability-based interaction design, including large fonts, high contrast, step-by-step guidance, slower interaction timing, tolerance for reduced movement, and configurable default channels.

**Hardware constraints in biosignal and advanced interaction channels.** Advanced interaction channels, such as sEMG and brain–computer interface (BCI), remain constrained by signal quality, calibration burden, comfort, setup time, fatigue, and maintenance requirements. These constraints are especially relevant in older-adult deployment contexts, where ease of use and sustained comfort are as important as technical performance. Therefore, this study positions sEMG and BCI as special-purpose or backup channels for high-value commands rather than as primary channels for routine interaction.

**Privacy, data security, and user control.** Social robots may collect highly sensitive data, including facial images, voice recordings, behavioral patterns, physiological signals, and potentially neural signals. These data may reveal users’ routines, health conditions, and emotional states. Privacy and data security should therefore be addressed across the full data lifecycle, including collection, transmission, storage, processing, and deletion. Practical safeguards include data minimization, local or edge processing, retention limits, granular permission management, and user-facing controls that allow older users and caregivers to understand and adjust privacy settings.

**Model governance, bounded adaptation, and responsibility allocation.** Future social robots may become more adaptive through continual learning and model updates, but increased adaptivity also raises concerns about explainability, reversibility, and responsibility when errors occur. Future systems should support bounded adaptive learning, human oversight, auditability, consent mechanisms, and clear allocation of responsibility among developers, service providers, caregivers, and users. These safeguards are particularly important for safety alerts, emotionally sensitive interactions, and functions that may influence care-related decisions.

### 6.3. Design Implications from an EI-Informed Perspective

The findings of this study can be interpreted through an EI-informed perspective, but EI should be understood here as a conceptual lens rather than a directly measured variable. From this perspective, multimodal social-robot design can be organized around three support processes: perceiving emotion-related cues, responding in an empathic and context-sensitive manner, and providing low-risk regulation-oriented support.

This interpretation helps clarify the role allocation of different modalities. Voice and visual channels can support everyday communication, confirmation, and contextual interpretation. Affective interaction can support non-diagnostic adjustments, such as tone, pacing, reminder intensity, and light social prompts. Gesture, touch, sEMG, and BCI can provide fallback or accessibility-oriented channels in specific contexts where voice or visual interaction is less reliable.

The survey results suggest that respondents were relatively willing to engage in affective interaction despite lower awareness of the technical term. This pattern may indicate latent demand for emotionally responsive interaction. However, this finding should be interpreted as design-oriented evidence of perceived desirability rather than evidence that such systems improve emotional-health outcomes.

The predominance of respondents aged 45–64 also suggests that the results primarily reflect anticipatory expectations of prospective users in later life. This is useful for early-stage design planning, but future studies should involve more adults aged 65 and above and evaluate prototype use in real-world settings.

Overall, the EI-informed interpretation helps connect user preferences, multimodal role allocation, and conceptual system architecture. It provides a design rationale for future prototype development and evaluation, but it should not be treated as evidence of intervention effectiveness.

## 7. Conclusions

As the global population ages, social robots are increasingly discussed as potential tools to support later-life users’ daily living, social engagement, and emotional well-being. However, single-modality interaction may not fully address the diversity of sensory abilities, cognitive habits, technology familiarity, and contextual constraints among middle-aged and older users. In this context, the present study examined user awareness, willingness, perceived barriers, functional priorities, and modality-function correspondences across six interaction modalities: visual, voice, somatosensory, affective interaction, sEMG, and BCI.

Based on an exploratory questionnaire and the related literature, this study proposed a conceptual five-layer architecture for multimodal social robots in later-life support contexts. The architecture includes the Intelligent Perception Layer, Basic Network Layer, Data Processing Layer, Service Application Layer, and Control and Management Center. Rather than serving as a validated intervention model, the architecture is intended as a design-oriented scaffold for organizing multimodal interaction channels, service functions, and age-friendly design considerations. The findings suggest that respondents generally showed openness to multimodal interaction while also expressing concerns about voice robustness, visual privacy, the use of affective data, wearable comfort, and the learning burden associated with advanced interfaces. Functional priorities were concentrated in health-related support, emotional companionship, learning and entertainment, and daily assistance. These patterns indicate that future social robots for later-life support should combine practical utility with emotionally responsive and configurable interaction, while avoiding overreliance on any single modality.

From an EI-informed perspective, the study interprets user preferences and modality patterns as design-oriented indications for prioritizing interaction capabilities related to emotion perception, empathic responsiveness, and assistance for regulation. In particular, the observed willingness toward affective interaction is interpreted as latent demand for emotionally responsive robot behavior rather than as evidence of intervention effectiveness. Based on a comparative analysis of modality strengths and constraints, the findings suggest a configurable strategy in which voice serves as the primary channel, supported by visual confirmation, a gesture-based backup interaction, and affective interaction as an enrichment layer, while sEMG/BCI are positioned as special-purpose accessibility channels in constrained scenarios.

The study contributes by: (1) Translating user-side constraints, preferences, and age-related variability into actionable multimodal design considerations for older-adult-oriented social robots; (2) Proposing a modular, conceptual architecture that connects multimodal interaction design with EI-informed support functions under privacy, safety, and accessibility constraints; (3) Using conceptual robot mapping to illustrate how heterogeneous social-robot platforms can be described within a common design framework. These contributions are intended to support future prototype development, empirical evaluation, and framework refinement.

This study also has limitations: First, the sample was dominated by respondents aged 45–64, with relatively few participants aged 65 years or above; therefore, the results primarily reflect prospective later-life users rather than the broader older-adult population. Second, as a questionnaire-based study involving participants with varying educational backgrounds and levels of technology familiarity, some responses may have been affected by misunderstandings or inconsistent interpretations of technical terms, especially sEMG and BCI. Third, this study did not test a working prototype or evaluate emotional-health outcomes. Future research should recruit more current older-adult participants; develop and test prototypes in real-world settings; and evaluate usability, acceptance, perceived support, and sustained engagement over time.

## Figures and Tables

**Table 1:** Demographic Characteristics of Respondents.

Characteristic	Category	*N*	%
Gender	Male	86	43.2%
	Female	113	56.8%
Age Group	45–49	39	19.6%
	50–54	65	32.7%
	55–59	25	12.6%
	60–64	49	24.6%
	65+	21	10.6%
Education	Primary school or below	27	13.5%
	Junior high school	38	19.0%
	Senior high school/Vocational school	65	32.6%
	Junior college (Associate degree)	29	14.5%
	Bachelor’s degree	37	18.6%
	Master’s degree or above	9	4.5%
Marital status	Married	188	94.4%
	Divorced	7	3.5%
	Widowed	4	2.0%
Residence	Living alone	1	0.5%
	Living with spouse	183	91.9%
	Living with children	12	6.0%
	Living with relatives or friends	3	1.5%

**Table 2:** Architectural Mapping of Representative Social Robots.

Robot	Intelligent Perception	Data Processing	Application Layer	Age-Friendly
PARO	Tactile response; audio interaction; posture/behavioral response	Internal state and interaction response	Therapeutic companionship; emotional stimulation	Zoomorphic seal form; soft tactile interaction
Pepper	Camera; microphone; sonar; tablet; gesture interaction	Communication and interaction logs; navigation/spatial awareness	Social communication; entertainment; training/exercise support	Humanoid form; multimodal feedback through voice, tablet, and gesture
ElliQ	Voice interaction; screen/touch interface; camera-supported interaction	Daily routine learning, wellness, and reminder data	Medication reminders; wellness guidance; aging-in-place companionship	Non-humanoid companion form; proactive prompts; simplified interaction
LOVOT	Thermal sensing; camera/depth sensing; tactile interaction	Presence detection; affective engagement cues	Emotional companionship; bonding; social presence	Warm body temperature; eye contact; soft embodied form
Mabu	Conversational interface; screen-based interaction; visual engagement	Patient self-report; symptom and medication-related data	Chronic-disease management; daily check-ins; health coaching	Personalized conversation; home-based healthcare companion
NAO	Speech; camera; gesture/movement; humanoid embodiment	Scenario-based interaction flow; response and engagement cues	Health-promotion education; social and cognitive engagement	Small humanoid form; demonstrative gestures; accessible verbal explanation

## Data Availability

The study is based on a questionnaire of older adults. The individual-level dataset contains potentially identifiable information (e.g., age, living arrangements, and usage preferences). Participants consented to research use with aggregate reporting, but not to public release of the raw records.

## Abbreviations

The following abbreviations are used in this manuscript:

AI	Artificial Intelligence
AU	Action Unit (facial muscle movement coding)
BCI	Brain–Computer Interface
EI	Emotional Intelligence
EEG	Electroencephalography
EMG	Electromyography
HCI	Human–Computer Interaction
HRI	Human–Robot Interaction
IoT	Internet of Things
LLM	Large Language Model
IMU	Inertial Measurement Unit
NLP	Natural Language Processing
RGB-D	Red-Green-Blue plus Depth
sEMG	Surface Electromyography
TAM	Technology Acceptance Model
TTS	Text-to-Speech
UI	User Interface

## Appendix A. Questionnaire

### Appendix A.1. Cover Note

Dear Participant,

Thank you for taking the time to complete this questionnaire.

With the rapid development of digital technologies such as artificial intelligence, social robots are gradually entering everyday life and show increasing potential in providing emotional companionship and daily support for older adults. This questionnaire aims to understand current and future preferences and needs regarding multimodal interaction in social robots for later-life support, in order to inform user-centered product design and related academic research.

Your participation in this survey is entirely voluntary. The questionnaire is anonymous and is intended for academic research purposes only. We do not collect personally identifiable information such as your name, phone number, ID number, exact address, or medical record information. All responses will be kept confidential and used only for research analysis.

Please answer the questions according to your own situation and understanding. Items marked with an asterisk (*) are required.

By proceeding to complete this questionnaire, you confirm that you have read the above information and voluntarily agree to participate in this study.

### Appendix A.2. Part I: Demographics

**Q1. Sex [Single choice] ***

○ Male ○ Female

**Q2. Age [Single choice] ***

○ 45–49 ○ 50–54 ○ 55–59 ○ 60–64 ○ 65+

**Q3. Marital status [Single choice] ***

○ Single ○ Married ○ Divorced ○ Widowed ○ Other: ______

**Q4. Education [Single choice] ***

○ Primary school or below ○ Junior high school

○ Senior high school/Vocational school

○ Junior college (Associate degree)

○ Bachelor’s degree ○ Master’s degree or above

**Q5. Current living arrangement [Single choice] ***

○ Living alone ○ Living with spouse ○ Living with children

○ Living with relatives/friends ○ Nursing home/senior care facility

**Q6. Employment status [Single choice] ***

○ Employed (go to Q7)

○ Self-employed (skip to Q8)

○ Retired (skip to Q8)

○ Retired—reemployed (skip to Q8)

○ Not currently working (unemployed/between jobs) (skip to Q8)

○ Other: ______ (skip to Q8)

**Q7. If you are currently employed, what is your occupation? [Single choice] ***

○ Marketing/Sales/Business ○ Procurement ○ Administration

○ Human resources ○ Product/Operations ○ Self-employed

○ Finance/Accounting/Cashier/Audit ○ Corporate management

○ Legal counsel/Lawyer ○ Designer

○ Service industry staff ○ R&D/Engineer

○ Agriculture/Forestry/Animal husbandry/Fishery worker

○ Manual worker ○ Teacher ○ Medical staff

○ Researcher ○ Government/Party & state organs

(After Q7, continue to Q8.)

**Q8. Approximate monthly income [Single choice] ***

○ No income ○ <RMB 1000 ○ RMB 1000–2999

○ RMB 3000–4999 ○ RMB 5000–7999

○ RMB 8000–9999 ○ ≥RMB 10,000

### Appendix A.3. Part II: Preferences for Multimodal Social Robots

**Q9. Have you heard of “multimodal interaction”? [Single choice] ***

(Interacting with a robot using multiple channels such as speaking, seeing, and body movements.)

○ Very familiar ○ Somewhat familiar ○ Heard of it

○ Heard of it but not clear ○ Never heard of it

**Q10. Which of the following interaction types have you heard of or understood? [Multiple choice] ***

□ Visual interaction—uses cameras and visual sensors to capture actions/facial expressions for image-based communication (e.g., robot detects a smile and plays upbeat music).

□ Speech/voice interaction—uses microphones and ASR to converse in natural language (e.g., user asks “today’s weather”; robot replies with real-time weather).

□ Affective interaction—infers user emotion from prosody and facial expressions and responds accordingly (e.g., comforting tone when user sounds upset).

□ Gesture interaction—recognizes hand/arm gestures as commands (e.g., “OK” gesture confirms a task).

□ Electromyographic interaction (sEMG)—skin electrodes detect muscle electrical signals to control the robot with subtle movements (e.g., finger micro-movement switches play mode).

□ Brain–computer interface (BCI)—sensors read brain activity and translate intentions into commands (e.g., imagining “forward” makes the robot move forward).

□ None of the above.

**Q11. If you were to interact with a robot, which interaction types would you want it to support? [Multiple choice] ***

(Definitions as in Q10.)

□ Visual interaction □ Speech/voice interaction □ Affective interaction

□ Gesture interaction □ sEMG interaction □ BCI interaction

**Q12. Desired functions—Healthcare & Caregiving [Multiple choice]**

□ Vital signs monitoring: heart rate, blood pressure, blood glucose; automatic alert on abnormality.

□ Medication reminder: schedule doses per physician’s advice, remind via voice/vibration.

□ SOS emergency call: one-button call to family/emergency services for falls or acute illness.

□ Rehabilitation training: voice guidance or body-interaction (e.g., gesture) for rehab exercises.

□ Health data management: record/analyze health data and generate reports for clinicians.

□ Other: ______

**Q13. Desired functions—Household Assistance [Multiple choice]**

□ Home appliance control: voice control of lights, air-conditioner temperature, etc.

□ Daily reminders: schedules (shopping, follow-up visits), weather forecast, news broadcast.

□ Navigation & wayfinding: indoor/outdoor positioning to locate items or destinations.

□ Remote assistance: family can check activity status via phone or converse through the robot.

□ Other: ______

**Q14. Desired functions—Education & Learning [Multiple choice]**

□ News broadcasting: periodic audio/text updates on current affairs of interest.

□ Hobby support: recommend online/offline activities (e.g., calligraphy, photography, opera).

□ Knowledge learning: courses on history/culture/health/technology with voice Q&A.

□ Skills training: cooking, handicraft, smartphone use, etc.

□ Cognitive training: memory games and logic tasks to slow cognitive decline.

□ Language learning: multi-language teaching; learn a new language or dialect.

□ Other: ______

**Q15. Desired functions—Entertainment & Interaction [Multiple choice]**

□ Audio-visual entertainment: music, opera, films; voice on-demand supported.

□ Social interaction: video calls with family/friends; virtual social activities (e.g., dance livestreams).

□ Game companionship: chess, mahjong, casual puzzle games.

□ Personalized recommendation: content tailored to preferences (e.g., documentaries, health talks).

□ Other: ______

**Q16. Desired functions—Security & Monitoring [Multiple choice]**

□ Home security: detect strangers or abnormal events via cameras/smart doors & windows.

□ Environmental monitoring: smoke and gas leak detection with timely alerts.

□ Anti-lost positioning: GPS/indoor localization to track location.

□ Night monitoring: infrared sensing at night; auto night-light and safety reminders.

□ Abnormal behavior detection: analyze activity patterns; detect long inactivity or falls.

□ Other: ______

**Q17. Desired functions—Emotional & Psychological Support [Multiple choice]**

□ Emotional listening: open-ended conversation to relieve loneliness.

□ Psychological counseling: mindfulness/relaxation; referral to a professional counselor.

□ Memory activation: music/photos from earlier years to evoke positive memories.

□ Festival greetings: greetings on birthdays and major festivals.

□ Personalized companionship: learn habits; proactively initiate topics (e.g., “What story today?”).

□ Other: ______

**Q18. Willingness to purchase/use a social robot in the future [Single choice] ***

○ Very unwilling ○ Unwilling ○ Neutral ○ Willing ○ Very willing

**Q19. If purchasing a social robot in the future, which aspects matter most to you? [Multiple choice] ***

□ Price □ Technical reliability □ Appearance/design □ Practical functions

□ User reputation □ Product quality □ Maintenance/service

□ Ease of use □ Other: ______

**Q20. If using a social robot in the future, which performance attributes do you value most? [Multiple choice] ***

□ Response speed □ Content accuracy □ Privacy protection □ Ease of operation

□ Natural interaction □ Emotional expressiveness □ Speech clarity

□ System stability (smooth, not prone to freeze/crash) □ Battery life

□ Elderly-friendly design (large fonts, moderate speech rate, simple buttons)

□ Other: ______

### Appendix A.4. Part III: Open-Ended Feedback

**Q21. What expectations do you have for future social robots? [Open text]**

**Q22. Any suggestions for this study? [Open text]**

*Skip logic note: Respondents who chose “Employed” in Q6 answer Q7 and then continue to Q8; all other Q6 options skip directly to Q8.*
